# Supplementary material for: Fungal Strain Influences Thermal Conductivity, Hydrophobicity, Color Homogeneity, and Mold Contamination of Mycelial Composites
Source: Materials (Basel). 2024 Dec 11;17(24):6050. doi: 10.3390/ma17246050 (PMC11727980; doi:10.3390/ma17246050)
Supplement: Supplementary file 1 [file materials-17-06050-s001.zip › materials-3350653-supplementary.pdf]

Joris Verhelst [1], Simon Vandersanden [1], Olivier Nouwen [1] and François Rineau [1,*]

[1] Environmental Biology, Centre for Environmental Sciences, Hasselt University, Diepenbeek, Belgium.

* Correspondence: francois.rineau@uhasselt.be; Tel.: +32(0)11 26 85 88

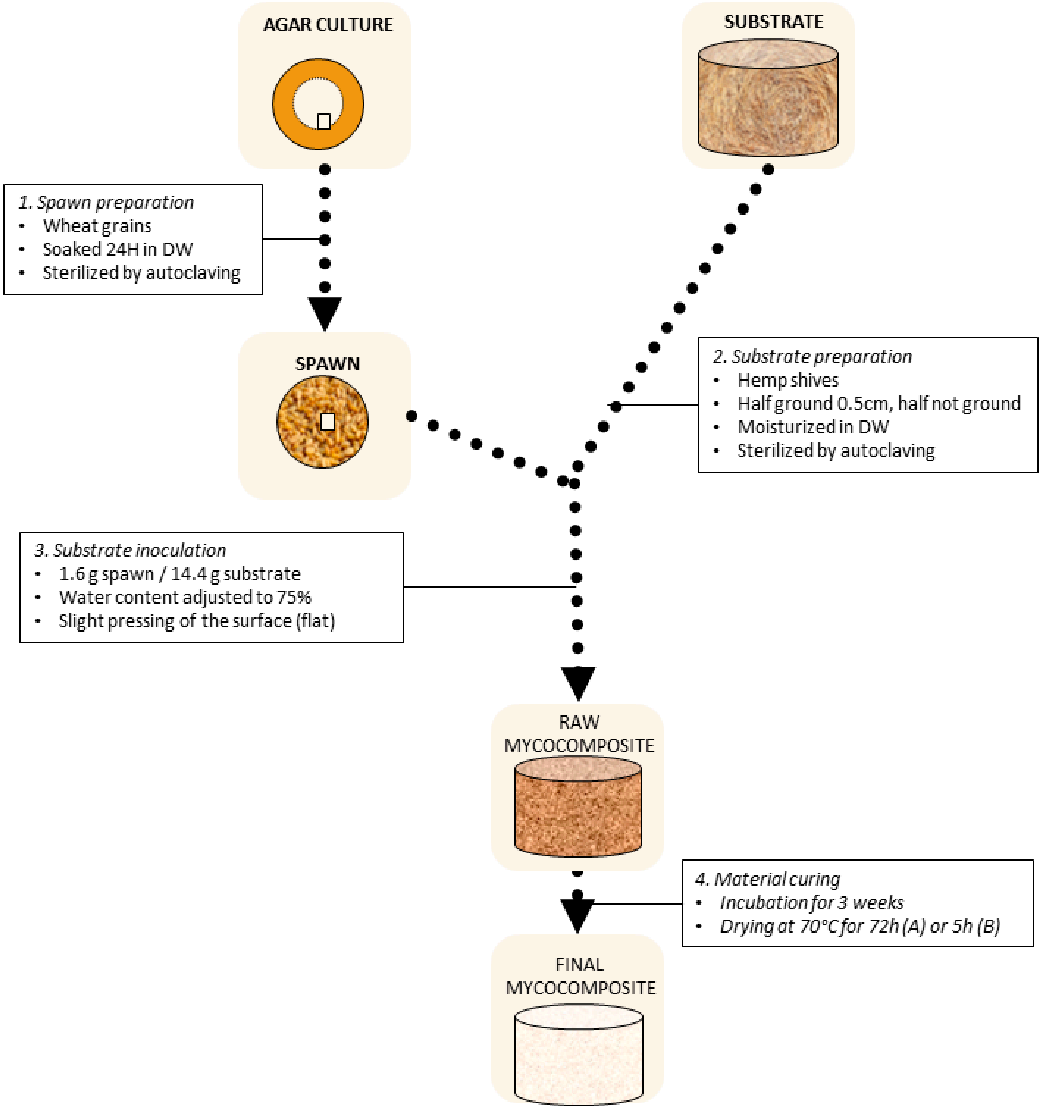

Figure S1. Graphical summary of the preparation of the composite materials.

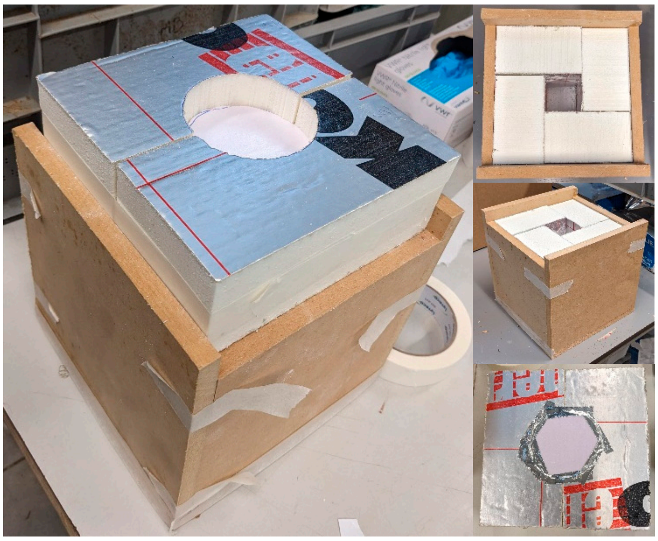

Figure S2: testing setup for insulation test. An insulated wooden box (17cm x 17cm x 17cm, inside dimensions) was insulated from the interior with 6cm thick XPS. The lid, made from the same XPS, was cut in two halves with a hole in the middle matching the dimensions of the materials to be tested (r=3.5cm). Samples were placed inside this lid hole and contact surfaces between sample and lid were sealed off with insulation tape. A temperature sensor was fitted in the beam shaped hole in the middle of the box.

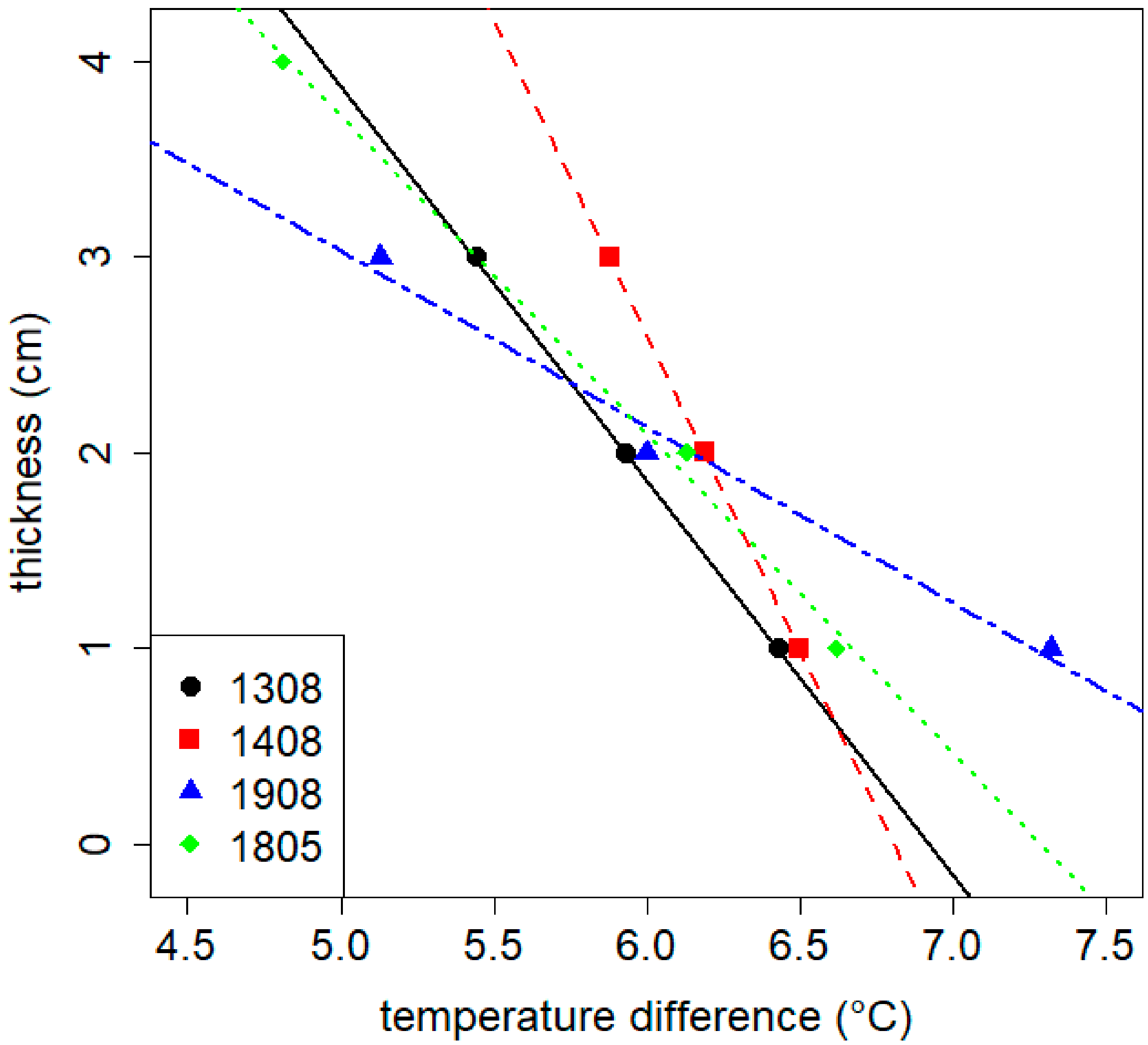

Figure S3: calibration curves constructed with three thicknesses (either 1, 2 and 3 cm or 2, 3 and 4cm) of XPS on four days used to calculate the thermal performance of the mycelial composites. Pearson's correlation for each calibration curve: rho = -0.999 and p = 0.003713 (1308), rho = -1 and p < 2.2e-16, rho = -0.993 (1408) and p = 0.07517 (1908), rho = -0.9975 and p = 0.04373 (1805)

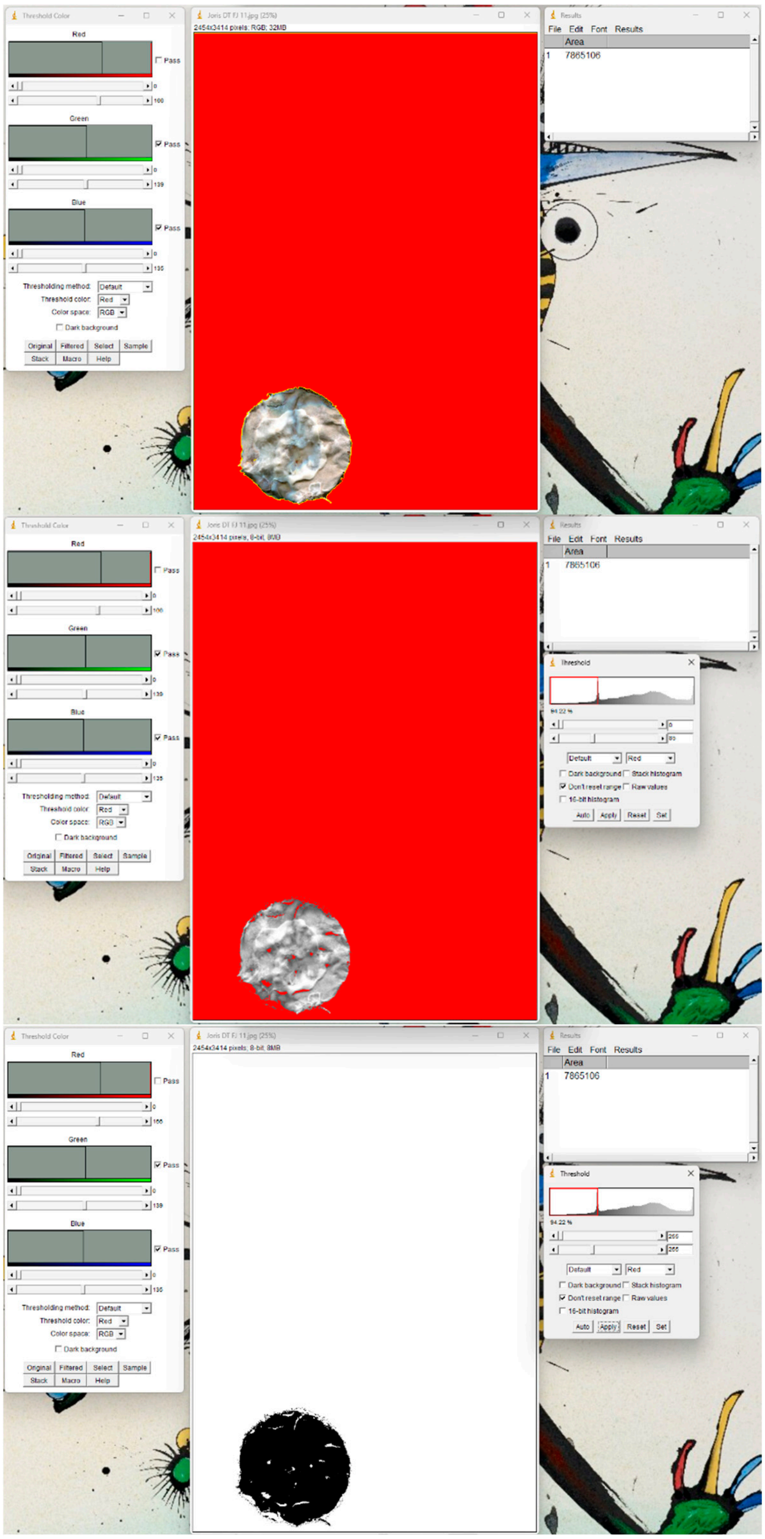

Figure S4: screenshots of the process to determine color evenness in imageJ

Table S1: min, mean, max and variance in cm XPS/cm material for mycelial composites. Values in cm XPS/cm material are then converted into W/(mK) using a value of 0.035 W/(mK) for XPS.

**Strain**	**Min (cm XPS/ cm material)**	**Mean (cm XPS/ cm material)**	**Max (cm XPS/ cm material)**	**Variance**	**Mean thermal conductivity converted in W/(mK)**
T. versicolor	0.55	0.9	1.35	0.07	0.039
D. tricolor	1.09	1.75	2.8	0.54	0.020
S. hirsutum	0.54	1.37	1.99	0.28	0.026
B. adusta	1.07	1.95	4.77	1.63	0.018
P. ignarius	0.9	1.26	1.77	0.1	0.028
F. fomentarius	0.61	0.88	1.07	0.04	0.040
UH-M	0.87	1.17	1.45	0.05	0.030
H. erinaceum	0.71	1.61	3.19	0.6	0.022
G. lucidum	-0.011	0.93	1.37	0.21	0.038

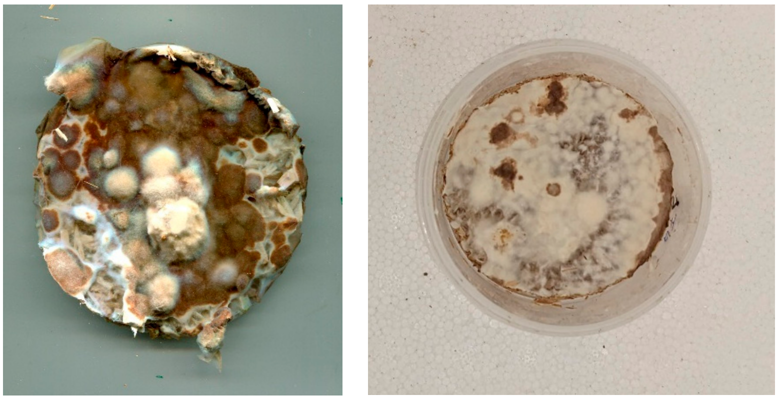

Figure S5: materials made with D. tricolor after four (left) and three (right) weeks of growth

Table S2: significant differences in the ratio of the surface area of mycelial composites covered in visible Trichoderma spores after 6 days (starting on day 32) of incubation at 30°C, 90% RH as observed with Dunn's test for experiment A and B in the treatment (T) group, exposed to an autoclaved solution of Trichoderma *propagules (1.6e5 on one cm²) in distilled water on day 25, 27 and 29 during growth and the control (C) group that was exposed to an equal volume of autoclaved distilled water*

**Strain**	**Strain**	**P – value**	**Group**	**Experiment**
GA	UHM	0.005283102	C	A
GL	UHM	0.049154091	C	A
DT	SH	0.0132681	T	B
SH	TV	0.01326281	T	B

Table S3: results of Mann-Whitney-Wilcoxon test (mean x > mean y) for significant differences between treatment groups for different strains as found significant in Dunn's test.

**Strain**	**C-CO (CO greater than C)**	**C-T (C greater than T)**	**CO-T (CO greater than T)**
BA	0.001082		
GA			0.002165
GL			0.001082
HE	0.004762		0.001082
PI			

UHM	0.001082		
DT			
TV		0.007937	
SH	0.01054		

Table S4: Comparison of thermal conductivity of the generated materials and most common, commercially available alternatives.

**Material**	**Range in thermal conductivity (W/(mK))**	
Mycelial composites (this study)	0.018	0.040
Mycelial composites (other studies)	0.041	0.104
XPS	0.029	0.037
PUR	0.022	0.028
Stone wool	0.035	0.045
Cellulose	0.038	0.040
Cork	0.038	0.045
Wood fibers	0.038	0.050
Glass wool	0.030	0.040
Hemp shives	0.050	0.060
